# Analysis of Coinfection Pathogens From Foot-and-Mouth Disease Virus Persistently Infected Cattle Using Oxford Nanopore Sequencing

**DOI:** 10.1155/2024/9703014

**Published:** 2024-09-18

**Authors:** Shuang Wang, Sumin Wei, Yaozhong Ding, Yun Zhang, Zhihui Zhang, Shiqi Sun, Huichen Guo, Shuanghui Yin

**Affiliations:** ^1^ State Key Laboratory for Animal Disease Control and Prevention College of Veterinary Medicine Lanzhou University Lanzhou Veterinary Research Institute Chinese Academy of Agricultural Sciences, Lanzhou, China; ^2^ Gansu Province Research Center for Basic Disciplines of Pathogen Biology, Lanzhou, China

## Abstract

The persistent infection caused by foot-and-mouth disease virus (FMDV) still lacks a reliable explanation, as its etiology and maintenance are intricate and potentially involve concurrent infections with multiple pathogens. In this study, we utilized the nanopore platform for direct sequencing of clinical samples obtained from cattle persistently infected with FMDV serotype O and investigated the distribution characteristics of coinfecting pathogens in their pharyngeal region. Briefly, we exploited Oxford Nanopore sequencing technology to generate high-quality and sufficient sequence data for the comprehensive characterization of microbial genomes. Furthermore, we performed sequence comparison, alignment, and phylogenetic tree construction. Our findings revealed a total of 23 viruses in FMDV carrier bovine, with FMDV, bovine orthopneumovirus, and *Choristoneura fumiferana* granulovirus emerging as the top three identified pathogens. The analysis unexpectedly revealed the presence of porcine circovirus type 2 and pepper mild mottle virus among the viral genes detected in the bovine FMDV carrier. Compared to noncarrier, carrier bovine of FMDV exhibited a greater diversity and abundance of mycoplasma types as well as reads counts. Therefore, we propose that the establishment and perpetuation of persistent FMDV infection may be attributed to the simultaneous presence of other viral agents and mycoplasmas. These findings highlight the significance of investigating multipathogen coinfection in elucidating the etiology of persistent FMD virus infection.

## 1. Introduction

Foot-and-mouth disease virus (FMDV; aphthovirus, *Picornacirus*) is the etiological agent of FMD and represents the most economically important ailment in the livestock industry [1]. FMDV primarily infects animals via aerosol transmission in the upper respiratory tract, specifically targeting the pharyngeal region [2]. FMD outbreaks result in immediate consequences, including mortality and reduced productivity. Additionally, some animals can become viral “carriers”, exhibiting persistent infections of FMDV in the saliva (esophageal–pharyngeal fluid, OPF). These carriers may include cattle, sheep, or goats, and viable viruses can still be isolated from their pharyngeal region beyond 28 days after infection [3, 4].

Carriers can develop even in vaccinated ruminants that have been exposed to the live virus, either during clinical or subclinical disease [3, 5]. The deposition of oropharyngeal fluid from persistently infected cattle into the nasopharynx of susceptible cattle has been experimentally demonstrated to be a causative factor for FMD [6]. Horizontal transmission in the persistent phase has only been demonstrated to occur among African Cape buffalo (*Syncerus caffer*) [6, 7]. Due to concerns regarding the potential transmission risk posed by carriers, authorities have implemented prolonged trade restrictions for animals and animal products following FMD outbreaks and from FMD-endemic regions [8]. Therefore, it is imperative to understand the status of animals persistently infected with FMD during the prevention of FMD outbreaks.

Samples of OPF taken from the pharynx are utilized for the analysis of FMDV in carriers, and they are essentially identical to those collected more than 28 days after infection [9]. *In situ* hybridization studies have shown that certain epithelial cells of the pharynx are positive for FMDV genomic material. Moreover, these cells can be cultured and remain virus-positive [10]. This finding suggested that the pharynx is likely the target region involved in persistent infection in cattle. The cause and maintenance of persistent infection are intricate and potentially involve coinfection by multiple pathogens [11]. The dynamics of coinfection can have profound consequences for disease transmission rates between hosts. Competition and transmission rates are closely linked within hosts, and differences in genotype arrival time can affect colonization [12]. The outcome may also be influenced by the host's immune response to the first pathogen. Therefore, comprehending within-host interactions and their impact on disease dynamics at the population level is crucial [13–15]. Furthermore, there is limited knowledge regarding spatial variation in coinfection levels across host populations [16].

Currently, the sequencing of FMDV is heavily reliant on the cumbersome process of transporting samples to well-equipped laboratories, leading to delays in responding to outbreaks. The Oxford Nanopore Technologies MinION portable sequencer is a viable solution because it enables sequencing to be performed in decentralized, remote laboratories located closer to outbreak locations. Traditional sequencing platforms used for FMDV characterization are typically limited to sophisticated facilities due to their intricate sample preparation protocols, costly equipment, and high computational requirements. While these platforms offer precise sequence analysis of samples, the necessity for sample transportation can compromise sample integrity and hinder swift decision-making in disease management. This challenge is particularly pronounced in regions endemic to FMDV, where limited resources and logistical obstacles impede the timely delivery of samples to laboratories. Therefore, the cost-effective and rapid sequencing capabilities offered by Oxford Nanopore sequencing have the potential to enhance FMD surveillance and control initiatives. This technology can aid in identifying circulating strains, thereby facilitating the development and deployment of appropriately matched vaccines in countries progressing along the FMD control continuum [17, 18].

Additionally, as a long-read sequencing technology, Oxford Nanopore sequencing offers maximum read lengths that significantly exceed those of viral genomes, positioning it as a leading method in the field of viral genome sequencing. Additionally, the extended read lengths enhance data assembly, particularly for pathogens with genomes characterized by a high number of repeats or complex structures. Unlike other sequencing platforms, Oxford Nanopore sequencing is the only technology capable of directly sequencing RNA and detecting base modifications. This unique advantage eliminates the biases and potential mutations associated with the reverse transcription of RNA into DNA for amplification when sequencing and studying RNA viruses [19].

In this study, we employed Oxford Nanopore sequencing technology to investigate the distribution characteristics of coinfection pathogens in the pharynx of cattle persistently infected with FMDV serotype O. The obtained results provide insights for further exploring the causes of persistent FMDV infection from the perspective of multipathogen coinfection.

## 2. Materials and Methods

### 2.1. Experimental Animals and Samples

The experiment was carried out within facilities at the Lanzhou Veterinary Research Institute in accordance with national animal welfare regulations. The experimental protocol for the cattle was approved by the Laboratory Animal Ethics Committee.

A total of 35 scalpers, immunized with Type O virus-like particle vaccine for 21 days, were challenged with FMDV/O/MYAY98/BY2001 through subcutaneous injection on the tongue. The animals were monitored daily, and blood, saliva, OPF, and nose swabs were collected over 35 days. At 28 days postinfection (dpi), two animals were identified as having a persistent infection, as FMDV nucleic acid was detected in the OPF. These two scalpers were euthanized at 35 dpi, and an autopsy was performed. Standardized samples, including the muzzle, oral mucosa, tongue, interdigital skin, pharynx tissue (the location where the OPF was collected), heart, lung, spleen, liver, kidney, and jejunum, were collected for further study.

### 2.2. RNA Extraction, Purification, and Amplification

RNA extraction from the pharyngeal tissue samples was carried out using the RNeasy Mini Kit (Qiagen, UK), following the manufacturer's protocol. The RNA was then cleaned and concentrated using the RNA Clean & Concentrator-5™ Kit (Zymo Research) for increased throughput. The purified RNA was amplified by reverse transcription with primer A (5′-GTT TCC CAC TGG AGG ATA-(N9)-3′). The PCR amplification was performed with random primer B (5′-GTT TCC CAC TGG AGG ATA-3′). PCR products were cleaned using AMPure XP Beads (Beckman) at a 1:1 ratio and then stored at −80°C until further use.

### 2.3. Libraries Preparation and MinION Sequencing

For sequencing library preparation, the Ligation Sequencing Kit and Native Barcoding Expansion 1-12/13-24 were utilized according to the manufacturer's instructions. The 50 fmol final libraries with adaptors were loaded onto an R90.4 flow cell (FLO-MIN 106D) and sequenced on the MinION Mk1C device for 8 h. ONT MinKNOW software (version 19.05.0) was used to collect the raw sequencing data, while ONT's cloud-based basecaller, based on Guppy (version 3.2.8), was utilized for onsite and real-time basecalling during the sequencing runs.

Validation of the identified viruses. RT-PCR was conducted using primers known to confirm the virus-specific presence of FMDV in the samples. The amplified products from RT-PCR were then sequenced, and the obtained sequences were further confirmed for the presence of each virus using a basic local alignment search tool (BLAST) search.

### 2.4. Data Analysis

Briefly, the demultiplexing of FASTQ reads was performed using PilotEdit Lite software (version 17.5.0). Subsequently, Minikraken2 was used to align the reads to a reference sequence generated by BLAST. The Wilbur-Lipman method within DNASTAR Lasergene MegAlign (version 7.1.0) was utilized for visualizing and comparing each consensus sequence with its respective reference sequence generated from BLAST to determine consensus accuracy. The ClustalW Multiple alignment within MEGA (version 11.0.13) was applied to construct a phylogenetic tree.

## 3. Results

### 3.1. Summary of Nanopore Sequencing Data

The utilization of Oxford Nanopore sequencing in the analysis of FMDV carrier and noncarrier bovine samples yielded a substantial amount of data, with 1,559,229 reads for carrier and 8,631,858 reads for noncarrier (Tables 1 and 2). Moreover, an impressive proportion (> Q7 both 100%) of reads successfully met the quality cutoff criteria (Figure S1). For the bovine FMDV carrier sample, Oxford Nanopore sequencing generated reads ranging in length from 1 to 8000 bp (Figure S2a). The majority of the reads (95%) were less than 2000 bp in length, with lengths less than 1000 bp accounting for 55% of the total reads. For a sample of FMDV noncarrier bovine, Oxford Nanopore sequencing revealed reads ranging in length from 1 to 9000 bp (Figure S2b), with the majority of the reads (87%) being shorter than 1000 bp.

### 3.2. Species Abundance

**Figure 1:**
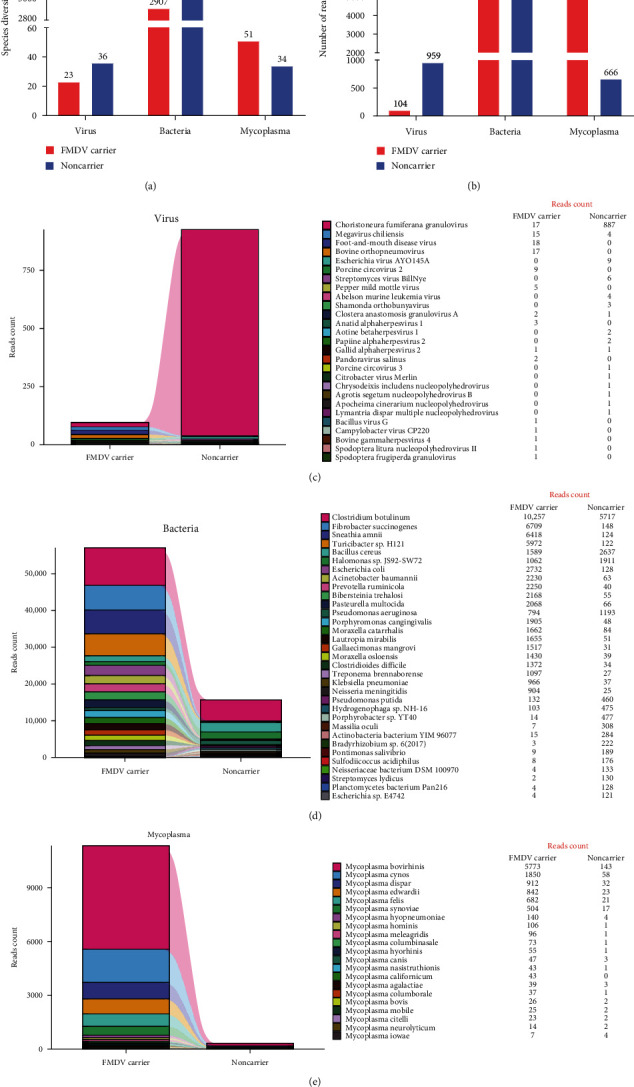
The taxonomic diversity and reads abundance of Oxford Nanopore sequencing data aligned to the reference genome of the target species: (a) species diversity, (b) species abundance, (c) virus, (d) bacteria, and (e) mycoplasma.

The reads obtained through Oxford Nanopore sequencing from bovine clinical samples, both FMDV carrier and noncarrier, were aligned to the reference genome of the respective species using Minikraken2, and the species diversity and abundance are indicated. In MinION sequencing, the reads count refers to the number of accurately measured microbe-specific read lengths. This metric is absolute in nature, making it the most direct indicator of microbe detection. By analyzing both the number of reads and the relative abundance of species, this technique enables semi-quantitative detection of microbial populations [20]. As depicted in Figure 1, a total of 104 reads aligned to 23 viruses were identified in the bovine FMDV carrier. Among these, the predominant viral species were FMDV, bovine orthopneumovirus (also known as bovine respiratory syncytial virus, BRSV), and *Choristoneura fumiferana* granulovirus. The noncarrier bovine sample generated a greater amount of data, with 36 viruses identified and a total reads count of 959 mapped to the reference species genome. Among these, the top three were *C. fumiferana* granulovirus, Escherichia virus AYO145A and Streptomyces virus BillNye. Moreover, 2907 bacteria with a total reads count of 286,065 were aligned in the bovine FMDV carrier. Among them, the predominant species were *Clostridium botulinum*, *Fibrobacter succinogenes*, and Sneathia amnii. The FMDV noncarrier bovine sample yielded a total reads count of 46,190, aligning with 3459 bacteria. Among these, the predominant species were *C. botulinum*, *Bacillus cereus*, and Halomonas sp. JS92-SW72. Additionally, the analysis revealed that a total of 51 mycoplasma species were identified in the bovine FMDV carrier, with a cumulative reads count of 25,912. Among these, the predominant species were *Mycoplasma bovirhinis*, *Mycoplasma cynos*, and *Mycoplasma dispar*. In addition, 34 mycoplasma species with a total reads count of 666 were aligned in FMDV noncarrier bovine, of which the top three were *M. bovirhinis*, *M. cynos*, and *M. dispar*.

### 3.3. Sequence Alignment

Following Oxford Nanopore sequencing, the reads were aligned against a reference database to identify the closest matching strain. A local sequencing database was constructed using BLAST for homology comparisons. The results were filtered, and only sequences with comparison scores exceeding 85 were retained as outcomes. Bovine orthopneumovirus (bovine respiratory syncytial virus, BRSV)-GCF-000849305.1-ViralProj14697-genomic (NC_001989.1), pepper mild mottle virus (PMMoV)-GCF-000859645.1-ViralProj15148-genomic (NC_003630.1), and porcine circovirus type 2 (PCV2)-GCF-002819625.1-ASM281962v1-genomic (NC_006232.1) were used as reference genomes for sequence alignment. Finally, 37 sequence fragments of BRSV were obtained through comparison (Table S1) and subsequently concatenated to generate 5 longer nonrepetitive sequence fragments.

**Figure 2:**
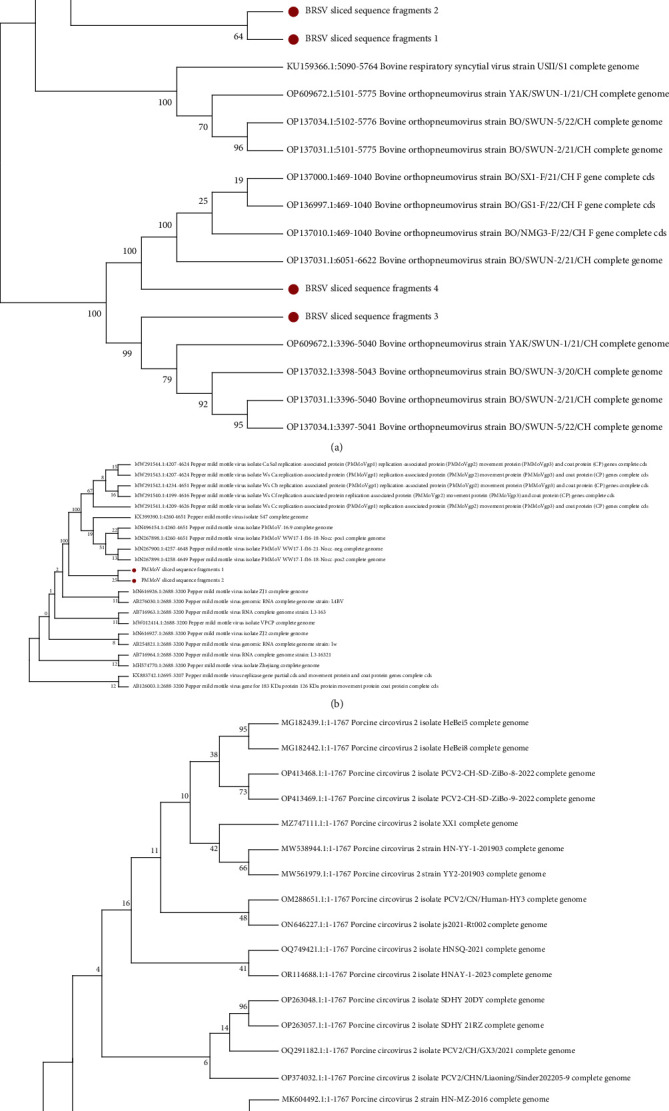
The phylogenetic tree was constructed based on the spliced sequence fragments: (a) bovine orthopneumovirus (bovine respiratory syncytial virus, BRSV), (b) pepper mild mottle virus (PMMoV), and (c) porcine circovirus 2 (PCV2).

Similarly, the 6 and 18 sequence fragments of PMMoV and PCV2 were obtained using the same method, and they were spliced to obtain 2 and 1 longer sequence fragments without repetition, respectively (Tables S2 and S3). Subsequently, a BLAST search was conducted to identify the top 20 most similar genome sequences, and a phylogenetic tree was constructed using MEGA software based on the ClustalW multiple alignment model (Figure 2). Bootstrapping of 1000 replicates was performed, and the numbers at the nodes represent percentage bootstrap support. The isolate names and GenBank accession numbers for the included sequences are provided in the trees. The alignment of complete genome sequences between the BRSV strain and our splice sequence fragments revealed the highest genetic relatedness with the BRSV strains BO/SWUN and YAK/SWUN in our findings. The PCV2 splice sequence fragments are closely related to those of strains isolated from PCV2MY-202012.

## 4. Discussion

FMDV can cause persistent infections for up to 5 years in African buffalo [21], 2 years in cattle [22], and 5–12 months in sheep and goats [23]. Vaccination with a homologous strain can elicit comparable antibody responses in serum and reduce the level of virus shedding during the acute stage of the disease, effectively preventing clinical disease in both carriers and noncarriers. However, it does not provide complete protection against subclinical or persistent infection [24]. The existence of a prolonged, asymptomatic carrier state poses a challenge to policymakers to the control and potential eradication of FMD. In free-FMD countries, particularly during FMD outbreaks, extensive culling of livestock is often implemented as a precautionary measure due to concerns regarding potential undiagnosed subclinical infections harbored by certain animals [25], despite uncertainties surrounding the biological significance of FMDV persistence. The implementation of such control measures would result in substantial economic losses for livestock farmers and increase meat product prices, thereby impacting the livelihoods of individuals. Since then, multiple studies have confirmed that the FMDV carrier state occurs in both nonvaccinated and vaccinated cattle. Nevertheless, the detailed mechanisms underlying the state of FMDV carriers, including the host–virus interactions that facilitate the long-term persistence of infectious viruses and the coevolution of viruses with the host immune system, have not been thoroughly elucidated.

Our current work aimed to investigate the distribution characteristics of coinfection pathogens in the pharynx of persistently infected cattle using Oxford Nanopore sequencing. A total of 23 viruses were detected in FMDV carrier bovine, with FMDV, BRSV, and *C. fumiferana* granulovirus being the three most commonly identified. In comparison, a total of 36 viruses were found in noncarrier bovine (Figure 1). Notably, our analysis unexpectedly revealed the presence of PCV2 and PMMoV among the viral genes detected in the FMDV carrier bovine.

A recent review suggested that cross-species transmission of PCVs might pose a serious threat to global animal industries [26]. In addition to pigs, the presence of serum antibodies against PCV2 has been detected in various animal species, including mice, cattle (both beef and calves), sheep, horses, and humans [27], indicating that these animals have been or are currently infected with PCV2. Three different genotypes (PCV2b, PCV2d, and PCV2e) were detected in buffalo [28]. An artificial infection experiment confirmed that PCV2 can infect cattle across the species barrier, and its clinical symptoms are similar to those of PCV2-infected pigs. The PCV2-infected cattle showed moderate clinical signs, including lymph node swelling, diarrhea, and reddening of the oral and ocular mucosa. Specific antibodies against PCV2 were detected in blood samples, while DNA was detected in various tissues [29]. Additionally, the PCV4 genome was detected in fecal samples of dairy cows [30]. In our study, the genome of the PCV2 isolate strain PCV2MY-202012 was matched in sample from cattle persistently infected with FMD. The findings of these studies provide further evidence supporting the potential cross-species transmission of PCV2 from pigs to cattle. Moreover, additional research is warranted to investigate whether PCV2 infection facilitates secondary infections caused by multiple pathogens.

To date, there is no evidence of shared hosts between plant viruses and vertebrate viruses [31]. Therefore, plant viruses have not been identified as pathogens for humans or other vertebrates, nor have they been shown to infect them [32]. Recent studies have shown that the boundaries between plants and animals might not be hermetical for plant viruses. PMMoV, a plant pathogen, is distributed worldwide and has been reported at the highest concentration in the feces of humans and a few animals [33]. After consuming processed food products containing infected peppers, PMMoV is often excreted in high concentrations in human feces. The presence of high concentrations of pathogens associated with human excreta in environmental waters or water supplies poses a significant threat to public health [34]. Subsequent investigations revealed a potential association between PMMoV infection and fever, abdominal pain, and pruritus in patients, which elicited specific immune responses. Notably, significant disparities were observed in clinical case statistics between individuals who were positive or negative for PMMoV, with anti-PMMoV IgM antibodies being detected in all PMMoV-positive patients [35]. This study revealed that plant viruses may have breached the limits of the “realm” to infect humans and even other mammals and cause clinical symptoms. In our study, the PMMoV genome was detected in samples from cattle with persistent FMDV infection. There is no further evidence to substantiate the viral infection status of the detected PMMoV genome. It is possible that this virus comes from drinking water or feed contaminated with the virus. However, our results suggested that PMMoV poses a potential risk of infection in mammals.

In the field of veterinary medicine, the prevalence of infectious diseases stemming from various causes is widespread. These complexities become apparent, particularly when mycoplasma and viruses are involved. Determining the individual impact of each agent in cases of coinfection or multiple etiologies becomes exceedingly challenging, if not entirely unfeasible, while also considering the cumulative adverse effects caused by these pathogens. Our research revealed significant differences in the types of pathogenic microorganisms present in persistently infected as opposed to noninfected FMD cattle, offering valuable insights for the investigation of ongoing FMDV infection. Compared to noncarrier, FMDV carrier cattle demonstrated a greater diversity and abundance of bacteria and mycoplasma types as well as reads counts (Figure 1). This indicates that FMDV carrier may exhibit an increased susceptibility to coinfection with bacteria and mycoplasma. Notably, viral diseases affecting cattle and poultry, especially those impacting the respiratory system, are often exacerbated by concurrent or subsequent mycoplasma infections [36]. The bovine respiratory epithelium possesses a remarkable ability to harbor various viral, bacterial, and mycoplasma pathogens owing to its intricate structural composition and susceptibility to environmental pollutants. Research has indicated the presence of multiple viral, bacterial, and mycoplasma pathogens in the respiratory tract of cattle, either as single infections or mixed infections [37]. *Mycoplasma bovis* is increasingly acknowledged as a significant pathogen despite lingering controversy in the field. The clinical manifestations attributed to *M. bovis* are nonspecific, rendering accurate economic assessments of its significance currently elusive [38]. The co-occurrence of *M. bovis* with other respiratory pathogens is frequent, highlighting its synergistic interplay with various bacterial or viral agents, such as bovine viral diarrhea virus and BRSV, in the progression of severe lesions.

Moreover, coinfection of mycoplasma family members is notably prevalent [39]. In this study, BRSV coinfection was detected in the oropharynx of cattle with persistent FMDV infection. Additionally, relatively high levels of *M. bovirhinis*, *M. cynos*, and *M. dispar* were detected. It is imperative to concentrate on coinfections and host responses to elucidate the underlying mechanisms of these potentially synergistic interactions. Previous studies have shown that influenza D virus (IDV) can increase cattle vulnerability to the respiratory pathogen *M. bovis* during coinfection. Specifically, IDV seems to impede the innate immune response against *M. bovis*, potentially facilitating increased proliferation of *M. bovis* and prolonging its presence in hosts [40, 41]. Nonetheless, the potential synergistic role of mycoplasma in the persistent FMDV infection remains unexplored, necessitating further investigation into the associated mechanisms.

Coinfections are common in nature and can significantly impact disease dynamics and severity. Despite this prevalence, there needs to be more studies assessing the interplay between a diverse pathogen community and persistent infection by FMDV. A previous study demonstrated that virus-induced lymphocyte proliferation or immunosuppression may increase susceptibility to additional virus replication and infection. Consequently, the influence of coinfecting pathogens on the development of FMDV persistence may stem from the impact of FMDV on the immune response of infected animals. On the one hand, FMDV infection targets immune cells, leading to immunosuppression and making the host more vulnerable to secondary or mixed infections with other pathogens. On the other hand, the presence of coinfection pathogens could impede the innate immune response triggered by FMDV. In the literature, comparable mechanisms have been documented. For example, IDV has been shown to suppress acute-phase response signaling and mitigate *M. bovis*-induced coagulation system activation when coinfection occurs, both of which play vital roles in the innate immune response [40]. Moreover, there is evidence suggesting that immune dysfunction induced by porcine parvovirus 7 may facilitate PCV2 replication in the context of coinfection [42]. However, further investigations into the fundamental mechanisms by which coinfecting pathogens interact with FMDV to impact viral persistence in carrier cattle will be crucial for elucidating this proposition.

The interplay between multiple coinfecting pathogens and FMDV could impact the efficacy of strategies employed to mitigate the prevalence of FMD within herds, notably vaccination. Impaired innate immunity linked to coinfecting pathogens might compromise the host's capacity to mount a robust immune response postvaccination. These insights underscore the importance of implementing holistic measures targeting bacteria, viruses, and mycoplasma for the prevention and management of FMDV, emphasizing the necessity of developing a multicomponent vaccine for preventing this disease.

In conclusion, the concurrent presence of other viral agents and mycoplasmas may influence the establishment and perpetuation of persistent FMDV infection. Our study provides insight into the etiology of persistent FMD virus infection by considering the phenomenon of multipathogen coinfection. Nevertheless, a definitive explanation for the persistence of FMDV-caused infections remains elusive, and the specific role of animals carrying the virus persistently in FMDV outbreaks remains uncertain. Accurately identifying animals carrying persistent FMD virus is paramount for preventing and managing FMD epidemics, especially in countries heavily reliant on vaccination as a primary preventive measure.

## Figures and Tables

**Table 1:** Basic characteristics of the bovine for each sample.

Sample	Breed	Gender	Age (year)	Weight (kg)
FMDV carrier	*Bos taurus domestica*	Male	1.0	149.0
Noncarrier	*B. taurus domestica*	Male	1.0	156.1

**Table 2:** Summary statistics of the Oxford Nanopore sequencing reads generated from each sample.

General summary	Mean read length	Mean read quality	Median read length	Median read quality	Number of reads	Read length N50	STDEV read length	Total bases
FMDV carrier	769.4	10.1	642.0	10.0	1,559,229.0	892.0	461.6	1,199,636,520.0
Noncarrier	517.3	10.0	478.0	10.1	8,631,858.0	527.0	231.9	4,465,331,041.0

## Data Availability

The data that support the findings of this study are available from the corresponding author upon reasonable request.

## Supporting Information

Supporting Information Table S1. Sequence fragments of BRSV based on reference genome alignment. Table S2. Sequence fragments of PMMoV based on reference genome alignment. Table S3. Sequence fragments of PCV2 based on reference genome alignment. Figure S1. Number and percentage of reads above the quality cutoff for Oxford Nanopore sequencing from each sample generated. Figure S2. Frequency distribution of Oxford Nanopore sequencing read lengths from each sample generated.
