# Supplementary material for: Analysis of Coinfection Pathogens From Foot-and-Mouth Disease Virus Persistently Infected Cattle Using Oxford Nanopore Sequencing
Source: Transbound Emerg Dis. 2024 Sep 18;2024:9703014. doi: 10.1155/2024/9703014 (PMC12016752; doi:10.1155/2024/9703014)
Supplement: Supporting Information — Table S1. Sequence fragments of BRSV based on reference genome alignment. Table S2. Sequence fragments of PMMoV based on reference genome alignment. Table S3. Sequence fragments of PCV2 based on reference genome alignment. Figure S1. Number and percentage of reads above the quality cutoff for Oxford Nanopore sequencing from each sample generated. Figure S2. Frequency distribution of Oxford Nanopore sequencing read lengths from each sample generated. [file 9703014.f1.docx]

## Supplementary Materials

**Table S1. Sequence fragments of BRSV based on reference genome alignment.**

| BRSV_ID | (Sequencing database) SD_ID | Identity | Alignment length | Mis  match | Gap open | BRSV start | BRSV end | SD start | SD end | Expect  value | Bit score |
| --- | --- | --- | --- | --- | --- | --- | --- | --- | --- | --- | --- |
| NC_001989.1 | 05afab9d-d426-4597-a67e-48e26877eee7 | 88.527 | 1229 | 94 | 35 | 3397 | 4595 | 1297 | 86 | 0 | 1466 |
| NC_001989.1 | 06e12355-d34b-4fb2-8f22-42f6fe424b6c | 94.706 | 170 | 4 | 4 | 2110 | 2275 | 261 | 93 | 7.07e^-64^ | 253 |
| NC_001989.1 | 13f0ad03-2394-47cb-9893-4b5ab4cef41c | 91.667 | 600 | 31 | 13 | 1561 | 2154 | 671 | 85 | 0 | 816 |
| NC_001989.1 | 162a1679-6537-467b-80dc-c466ea9f2641 | 89.886 | 613 | 32 | 21 | 1561 | 2149 | 91 | 697 | 0 | 759 |
| NC_001989.1 | 17520f4c-a91c-4bc6-902d-e51a55472bc2 | 91.343 | 335 | 16 | 10 | 14262 | 14584 | 417 | 84 | 2.46e^-120^ | 441 |
| NC_001989.1 | 17ed3d94-5c54-403b-aeb7-5098b2f303ee | 89.788 | 519 | 39 | 10 | 4529 | 5042 | 253 | 762 | 0 | 665 |
| NC_001989.1 | 1b15fd12-7f7f-4fcb-a68c-8741622d61ce | 85.385 | 130 | 13 | 4 | 5314 | 5440 | 92 | 218 | 1.37e^-28^ | 137 |
| NC_001989.1 | 1c4f2d85-82ef-4171-8622-94ff4ddd5eee | 92.679 | 642 | 22 | 17 | 10555 | 11182 | 95 | 725 | 0 | 893 |
| NC_001989.1 | 1d4e8f4a-2714-4ae9-8ecb-9f967d535d08 | 85.63 | 682 | 66 | 20 | 5114 | 5776 | 772 | 104 | 0 | 728 |
| NC_001989.1 | 21ffe327-2894-45c5-a4f4-9f7a748d08e6 | 92.647 | 340 | 14 | 6 | 10722 | 11058 | 83 | 414 | 2.30e^-133^ | 484 |
| NC_001989.1 | 315f93a7-272e-455c-826a-15613b5822c0 | 93.368 | 573 | 19 | 11 | 1025 | 1592 | 667 | 109 | 0 | 830 |
| NC_001989.1 | 3c1ae95a-f18c-4ed1-b6ff-9fca2ada73f0 | 92 | 325 | 17 | 7 | 2387 | 2708 | 101 | 419 | 5.79e^-122^ | 446 |
| NC_001989.1 | 5bae4bea-3fa3-4586-836d-e83740d6cfe4 | 87.574 | 169 | 9 | 6 | 6647 | 6809 | 233 | 395 | 5.81e^-46^ | 195 |
| NC_001989.1 | 5d60a9ba-0586-465c-9fda-21264d30ef0d | 96.296 | 54 | 1 | 1 | 14161 | 14214 | 392 | 340 | 2.17e^-13^ | 86 |
| NC_001989.1 | 68501a18-7b74-4f7d-bdbc-c12b8ebff7fa | 87.859 | 313 | 24 | 10 | 2528 | 2836 | 404 | 102 | 1.89e^-96^ | 361 |
| NC_001989.1 | 6f9c3e40-7e4e-49c6-81eb-fca54b39ec54 | 85.652 | 683 | 76 | 19 | 5103 | 5776 | 102 | 771 | 0 | 725 |
| NC_001989.1 | 7925e487-8da8-42bd-837a-9da15f0d8864 | 89.508 | 305 | 24 | 4 | 5335 | 5635 | 637 | 337 | 9.18e^-107^ | 396 |
| NC_001989.1 | 86933332-6dd7-42be-974c-e18559f22cbb | 89.39 | 377 | 19 | 11 | 1862 | 2228 | 105 | 470 | 5.07e^-129^ | 470 |
| NC_001989.1 | 889f3426-feaa-4a09-8595-ff086e310794 | 85.855 | 304 | 29 | 11 | 5335 | 5635 | 380 | 88 | 2.02e^-83^ | 318 |
| NC_001989.1 | 978b5b73-b602-448d-8af0-2b356f748c3d | 87.387 | 111 | 11 | 3 | 4926 | 5033 | 89 | 199 | 7.08e^-26^ | 127 |
| NC_001989.1 | 97dca26b-b74b-420f-8174-60ca4f5b128b | 92.857 | 70 | 3 | 2 | 5299 | 5366 | 1089 | 1020 | 1.20e^-16^ | 97.8 |
| NC_001989.1 | 988e365e-0a11-4841-9f43-5be566f1fc63 | 86.034 | 179 | 16 | 8 | 5265 | 5435 | 394 | 217 | 1.05e^-42^ | 183 |
| NC_001989.1 | 988e365e-0a11-4841-9f43-5be566f1fc63 | 89.6 | 125 | 10 | 2 | 5296 | 5417 | 217 | 93 | 3.43e^-36^ | 161 |
| NC_001989.1 | 9aea0454-a5a4-459b-974d-15ade811ad96 | 88.889 | 126 | 12 | 1 | 5321 | 5446 | 189 | 312 | 3.43e^-36^ | 162 |
| NC_001989.1 | a80e7124-1eaf-4f93-beb8-63aad484dc37 | 86.441 | 295 | 19 | 12 | 1994 | 2277 | 370 | 86 | 7.06e^-83^ | 317 |
| NC_001989.1 | a9bf90c4-8467-4cee-aff1-033204223c47 | 91.521 | 401 | 14 | 13 | 2205 | 2599 | 86 | 472 | 6.17e^-147^ | 530 |
| NC_001989.1 | a9c3c0db-b12f-43a3-9558-1ac6a8e1f9ee | 93.04 | 273 | 11 | 8 | 6149 | 6418 | 97 | 364 | 2.46e^-101^ | 379 |
| NC_001989.1 | ad3d86dc-0eda-4ca6-8c46-372397b4892e | 94.162 | 394 | 18 | 4 | 7739 | 8131 | 90 | 479 | 1.77e^-166^ | 594 |
| NC_001989.1 | b4d534ed-cb3a-4cf4-8324-dea848443209 | 90.252 | 595 | 32 | 19 | 1555 | 2135 | 669 | 87 | 0 | 750 |
| NC_001989.1 | babca341-a18c-4ae7-914a-cc1f99721a88 | 88.615 | 325 | 16 | 13 | 2528 | 2843 | 402 | 90 | 7.05e^-102^ | 380 |
| NC_001989.1 | be1db975-40f2-4cb6-bf4c-7007cdcb5d21 | 85.47 | 117 | 8 | 5 | 1882 | 1989 | 240 | 124 | 1.05e^-23^ | 121 |
| NC_001989.1 | c5a170eb-fe81-47b1-a6b1-c03a88eced5e | 90.323 | 124 | 10 | 2 | 5294 | 5416 | 212 | 90 | 9.83e^-37^ | 163 |
| NC_001989.1 | df5cd20b-bff9-492e-a7f1-1ed5a2ce6ae1 | 87.391 | 230 | 14 | 9 | 5550 | 5777 | 330 | 546 | 5.80e^-65^ | 258 |
| NC_001989.1 | e0d3872e-fe30-498f-af7a-0a53601ef9ff | 91.167 | 600 | 24 | 19 | 8919 | 9503 | 683 | 98 | 0 | 784 |
| NC_001989.1 | e57e95ad-d018-4c1f-bbcf-2a729142a359 | 90.646 | 588 | 32 | 17 | 6047 | 6624 | 76 | 650 | 0 | 757 |
| NC_001989.1 | f56fdd81-2001-4a1e-9e5d-b1315ae0b1b3 | 85.849 | 106 | 10 | 4 | 5314 | 5418 | 193 | 294 | 5.44e^-21^ | 111 |
| NC_001989.1 | fc199d9b-67bd-46ae-8138-6ee22450999e | 91.269 | 607 | 30 | 15 | 1560 | 2154 | 1322 | 727 | 0 | 810 |

**Table S2. Sequence fragments of PMMoV based on reference genome alignment.**

| PMMoV_ID | (Sequencing database) SD_ID | Identity | Alignment length | Mis  match | Gap open | PMMoV start | PMMoV end | SD start | SD end | Expect  value | Bit score |
| --- | --- | --- | --- | --- | --- | --- | --- | --- | --- | --- | --- |
| NC_003630.1 | 42865931-86c4-477b-834e-c708051fb212 | 88.416 | 423 | 17 | 17 | 4234 | 4651 | 483 | 88 | 5.34e^-137^ | 495 |
| NC_003630.1 | 68d7f5b6-19ce-4932-8f70-ac9132f8798e | 91.874 | 443 | 12 | 14 | 3887 | 4319 | 83 | 511 | 2.12e^-167^ | 596 |
| NC_003630.1 | 925e3e13-46f1-4840-b090-7b7476f92d71 | 88.587 | 552 | 26 | 24 | 2667 | 3200 | 81 | 613 | 6.93e^-180^ | 637 |
| NC_003630.1 | 97440351-e0e8-48f9-afd8-763d6f505cc9 | 86.631 | 561 | 38 | 26 | 2665 | 3200 | 638 | 90 | 9.02e^-166^ | 590 |
| NC_003630.1 | 9fc9806b-313b-4edc-85e8-b082aae4d9f9 | 90.233 | 430 | 18 | 16 | 4234 | 4651 | 505 | 88 | 1.74e^-149^ | 536 |
| NC_003630.1 | fdce7ff2-b416-4fbb-b6fb-875de8dc8541 | 91.304 | 161 | 8 | 6 | 179 | 335 | 87 | 245 | 3.86e^-50^ | 206 |

**Table S3. Sequence fragments of PCV2 based on reference genome alignment.**

| PCV_ID | (Sequencing database) SD_ID | Identity | Alignment length | Mis  match | Gap open | PCV2 start | PCV2 end | SD start | SD end | Expect  value | Bit score |
| --- | --- | --- | --- | --- | --- | --- | --- | --- | --- | --- | --- |
| NC_006232.1 | 15c59aa8-7cf2-4ea9-8c8a-0644f978b596 | 87.873 | 973 | 68 | 32 | 485 | 1442 | 1033 | 96 | 0 | 1124 |
| NC_006232.1 | 185f3c92-fc41-4c90-af93-0507151d5914 | 91.371 | 197 | 9 | 5 | 1335 | 1530 | 222 | 411 | 4.50e^-68^ | 264 |
| NC_006232.1 | 18acfe72-61fc-4e99-bd50-b507eea9c8dc | 87.132 | 544 | 42 | 21 | 1 | 528 | 540 | 1071 | 5.83e^-168^ | 597 |
| NC_006232.1 | 18acfe72-61fc-4e99-bd50-b507eea9c8dc | 90.196 | 459 | 28 | 14 | 1316 | 1767 | 91 | 539 | 1.56e^-162^ | 578 |
| NC_006232.1 | 3a1f9ca6-00a5-4a6e-8736-2cc8a4e9d447 | 86.973 | 261 | 20 | 12 | 1276 | 1530 | 928 | 676 | 7.13e^-72^ | 277 |
| NC_006232.1 | 4c1e0ece-73cf-4f04-a1ca-ce44c5144f7f | 85.28 | 856 | 63 | 36 | 618 | 1437 | 922 | 94 | 0 | 871 |
| NC_006232.1 | 6e24d037-d14f-4fa0-bac1-c833cfd2c0fd | 90.329 | 486 | 30 | 10 | 23 | 502 | 566 | 92 | 6.64e^-180^ | 636 |
| NC_006232.1 | 76d8dc5d-4586-41fe-b4d3-29cbaf162ef2 | 88.355 | 979 | 63 | 33 | 485 | 1442 | 463 | 1411 | 0 | 1150 |
| NC_006232.1 | 79389812-d85f-4967-8089-4ccd8b195cfd | 88.132 | 455 | 34 | 14 | 1317 | 1767 | 1067 | 629 | 5.83e^-149^ | 533 |
| NC_006232.1 | 79389812-d85f-4967-8089-4ccd8b195cfd | 91.386 | 534 | 34 | 12 | 1 | 527 | 628 | 100 | 0 | 713 |
| NC_006232.1 | 7ce421c6-1df0-4119-8bc8-4a58bb768f4a | 88.825 | 519 | 32 | 15 | 1 | 511 | 621 | 121 | 2.82e^-178^ | 631 |
| NC_006232.1 | 7ce421c6-1df0-4119-8bc8-4a58bb768f4a | 89.703 | 437 | 22 | 17 | 1340 | 1767 | 1044 | 622 | 7.10e^-148^ | 530 |
| NC_006232.1 | 8f128ac4-0405-4dd0-a205-b86cf6ee703b | 88.802 | 509 | 29 | 16 | 23 | 509 | 591 | 89 | 2.17e^-173^ | 615 |
| NC_006232.1 | 96c9749a-d5ae-4707-95ff-a73c1ac3e812 | 87.935 | 431 | 29 | 15 | 1343 | 1767 | 1039 | 626 | 4.20e^-138^ | 497 |
| NC_006232.1 | 96c9749a-d5ae-4707-95ff-a73c1ac3e812 | 89.239 | 539 | 31 | 18 | 1 | 528 | 625 | 103 | 0 | 655 |
| NC_006232.1 | b5677cdd-79ec-4a73-97cb-fbf4e7423cb2 | 86.029 | 272 | 21 | 8 | 1036 | 1298 | 95 | 358 | 2.18e^-78^ | 299 |
| NC_006232.1 | f13af34b-b54b-438b-8387-a86f4ec4bf1c | 87.419 | 461 | 33 | 18 | 1316 | 1765 | 85 | 531 | 1.91e^-142^ | 512 |
| NC_006232.1 | f1c86d73-503a-47d6-954c-bfd0127610fc | 90.795 | 478 | 26 | 13 | 23 | 496 | 89 | 552 | 1.46e^-175^ | 622 |


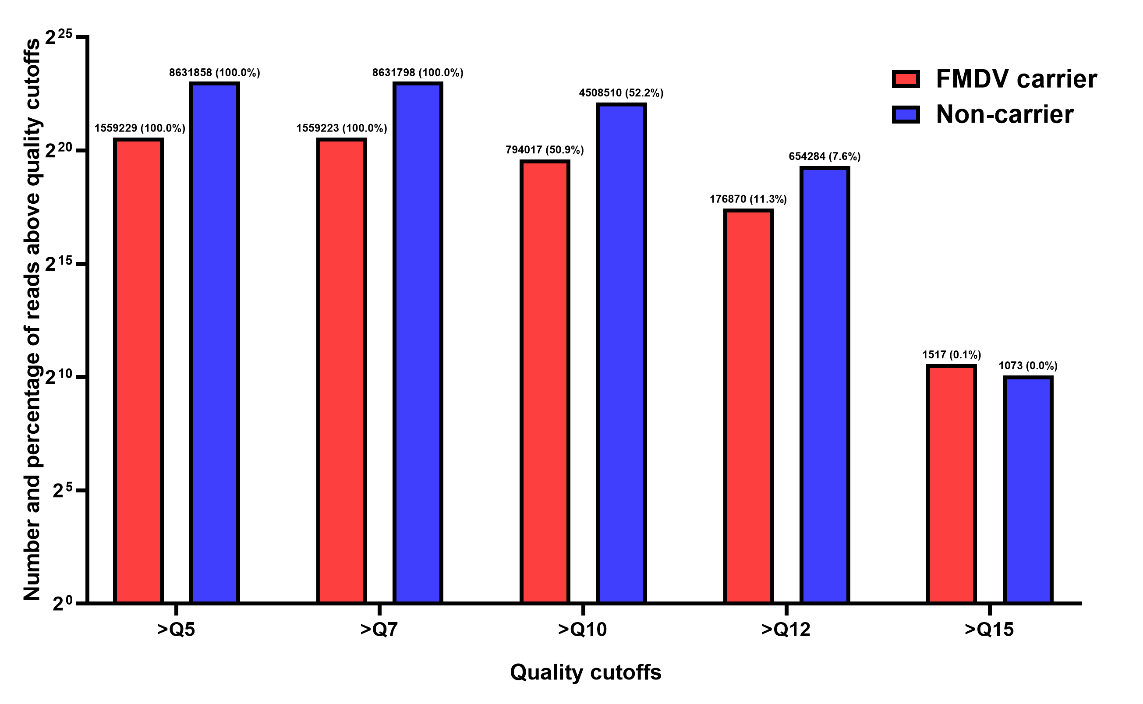


**Figure S1. Number and percentage of reads above the quality cutoff for nanopore sequencing from each sample generated.**


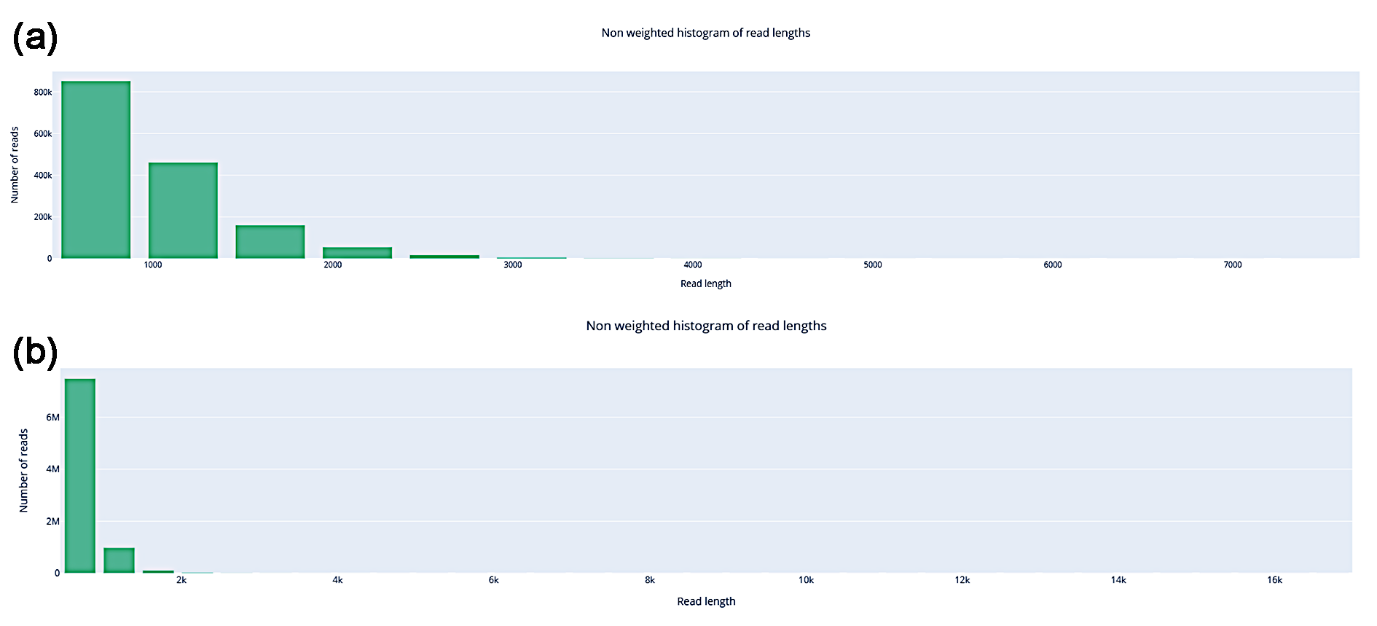


**Figure S2. Frequency distribution of nanopore sequencing read lengths from each sample generated.** (a) FMDV carrier; (b) FMDV noncarrier. (Read length unit: base pair, bp; M: million; k: kilo)
